# Interhemispheric characterization of small vessel disease imaging markers after subcortical infarct

**DOI:** 10.1002/brb3.595

**Published:** 2016-11-03

**Authors:** Maria del C. Valdés Hernández, Xinyi Qiu, Xin Wang, Stewart Wiseman, Eleni Sakka, Lucy C. Maconick, Fergus Doubal, Cathie L. M. Sudlow, Joanna M. Wardlaw

**Affiliations:** ^1^ Department of Neuroimaging SciencesCentre for Clinical Brain SciencesUniversity of EdinburghEdinburghUK; ^2^ College of Medicine and Veterinary MedicineUniversity of EdinburghEdinburghUK; ^3^ Buckinghamshire Healthcare NHS TrustBuckinghamshireUK

**Keywords:** magnetic resonance images, neuroimaging, small vessel disease, stroke, white matter hyperintensities

## Abstract

**Background:**

In structural Magnetic Resonance Imaging (MRI) of patients with a recent small subcortical infarct (RSSI) and small vessel disease (SVD) imaging markers coexist. However, their spatial distribution and prevalence with respect to the hemisphere of the RSSI remain unknown.

**Materials and Methods:**

From brain MRI in 187 patients with an acute lacunar ischemic stroke clinical syndrome and a relevant diffusion weighted imaging (DWI)‐positive lesion, we semiautomatically extracted the RSSI, microbleeds, lacunes, old cortical infarcts, and white matter hyperintensities (WMH) using optimized thresholding in the relevant sequences, and rated the load of perivascular spaces. We registered all images to an age‐relevant brain template and calculated the probability distribution of all SVD markers mentioned for patients who had the RSSI in each hemisphere separately. We used the Wilcoxon and chi‐squared tests to compare the volumes and frequencies of occurrence, respectively, of the SVD markers between hemispheres throughout the sample.

**Results:**

Fifty‐two percent patients (*n *= 97) had the RSSI in the left hemisphere, 42% (*n *= 78) in the right, 2.7% (*n *= 5) in both, and 3.7% (*n *= 7) in the cerebellum or brainstem. There was no significant difference in RSSI frequency between left and right hemispheres (*p *= .10) in the sample. The median volume of the RSSI (expressed as a percentage of the total intracranial volume) was 0.05% (IQR = 0.06). There was no difference in median percent volume of the right RSSIs versus left (*p *= .16). Neither was there a significant interhemispheric difference in the volume of any of the SVD markers regardless of the location of the RSSI and they were equally distributed in both hemispheres.

**Conclusion:**

Assessment of SVD imaging markers in the contralateral hemisphere could be used as a proxy for the SVD load in the whole brain to avoid contamination by the RSSI of the measurements, especially of WMH.

## 1 Introduction

Recent small subcortical infarcts (RSSI) may present with acute lacunar ischemic stroke symptoms (Wardlaw, Smith, Biessels, et al., 2013) which cause about 25% of all incident ischemic strokes, equivalent to about 35,000 patients per year in the United Kingdom (Bamford, Sandercock, Jones, & Warlow, 1987; Sacco et al., 2006). In structural MRI (Magnetic Resonance Images), RSSI is clinically associated with other imaging features of brain small vessel disease (SVD), including white matter hyperintensities (WMH), lacunes, perivascular spaces, and microbleeds (Wardlaw, Smith, & Dichgans, 2013). In brain MRI of patients with RSSI, these features coexist together with the acute lesion. In some cases, cortical hyperintense lesions indicative of previous cortical strokes, WMH, and recent stroke lesions coalesce together, making the correct delineation of their boundaries difficult, and sometimes subjective. Failure to correctly identify these features and discern between those that have similar signal intensities in MRI can result on a gross distortion of their load, having important implications for the design and sample size calculations of observational studies and randomized trials using WMH volume or WMH progression as outcome measures (Wang, Valdes Hernandez, Doubal, Chappell, & Wardlaw, 2012). Therefore, restricting the analysis of SVD imaging biomarkers to the contralateral hemisphere as a proxy of the SVD burden in the brain could be a useful approach. However, although some studies have adopted it assuming that these biomarkers are spatially distributed equally across both hemispheres (Lin et al., 2015); reports of their spatial prevalence with respect to the location of the RSSI differ.

Studies on the spatial distribution of microbleeds or lacunes (Duering et al., 2013; Lee et al., 2004; Sun et al., 2009) and WMH (Alexander et al., 2010; Del Bene, Makin, Doubal, Inzitari, & Wardlaw, 2013) are scant, and focused on describing and analyzing the spatial relationship between two biomarkers (Alexander et al., 2010; Del Bene et al., 2013; Duering et al., 2013; Lee et al., 2004) or the appearance and location of one of them on different pathologies (Lee et al., 2004; Sun et al., 2009). It has been suggested that the load of SVD biomarkers after an ischemic stroke could be greater in the hemisphere where the symptomatic lesion occurs. Endothelial dysfunction appears to be involved in the pathophysiology of SVD and indeed ischemic stroke (Wardlaw et al., 2009). Dysregulation of the endothelium results in a number of altered metabolic pathways including increased production of TGF‐β1 (transforming growth factor beta‐1) in ischemic conditions (Thompson & Hakim, 2009). When this occurs after stroke, the normal regulation of blood flow in the brain is thought to be altered, thus prompting ischemic conditions. A study found increased TGF‐β1 levels were prevalent in the ipsilateral hemisphere of the stroke, whereas very low levels were found in the contralateral hemisphere (Krupinski, Kumar, Kumar, & Kaluza, 1996). Another study found significant lower cerebral blood flow and cerebral metabolic rate of oxygen in the ipsilateral hemisphere as opposed to the contralateral hemisphere after stroke (Liu & Li, 2015). It has been also suggested that more lacunes could form in the ipsilateral hemisphere (Markus, 2004).

Thus, at present, there is insufficient evidence to support the assessment of SVD markers only in the contralateral hemisphere as a proxy for the SVD load in the whole brain. With this study, we aimed to seek evidence to underpin the selection of regions of interests to analyze biomarkers of SVD in the acute phase after a subcortical stroke by researching the distributional patterns of these biomarkers and their spatial relation to each other and with the RSSI. By means of an interhemispheral characterization of the SVD imaging markers on a relatively large group of stroke patients who had a visible RSSI identified in diffusion weighted imaging (DWI) and without any neuroradiological indication of a previous brain trauma, we want to inform on the feasibility of restricting the analysis of SVD imaging biomarkers to the contralateral hemisphere only as a proxy of the SVD burden in the brain, so as to avoid inaccuracies that could be caused by the presence of the RSSI.

## 2 Materials and Methods

### 2.1 Subjects and Brain MRI Acquisition

Brain MRI from three prospective nonoverlapping studies of patients with acute stroke was used. All patients presented to the same regional teaching hospital between: 2002 and 2005 (*n *= 87) (Study 1; Jackson et al., 2009), 2005 and 2007 (*n *= 32) (Study 2; Wardlaw et al., 2009), and 2010 and 2013 (*n *= 75) (Study 3; Munoz Maniega et al., 2016). Patients from Study 1 included all OCSP (Oxfordshire Community Stroke Project; Bamford, Sandercock, Dennis, Burn, & Warlow, 1991) ischemic stroke subtypes, whereas patients from Studies 2 and 3 included lacunar, partial anterior, and posterior circulation stroke syndromes only. However, we only included those who had presented with an acute lacunar stroke syndrome according to the OCSP classification and who had a corresponding RSSI visible on DWI. The mean age of the sample was 66.5 years (standard deviation 11.9, range 36–96). All patients were assessed by experienced stroke physicians, had their baseline demographics, full medical examination, vascular risk factors and other details recorded, and underwent routine stroke investigations (as described in Jackson et al., 2009; Wardlaw et al., 2009; Munoz Maniega et al., 2016). The diagnostic MRI scan was obtained in the interval between stroke onset and 4 weeks after. From a total of 194 patients, 113 (58%) were men, 62 (32%) had a previous stroke, 21 (11%) had diabetes, 121 (62%) had hypertension, and 32 (16%) had a previous history of cardiovascular disease. Written informed consent was obtained from all patients and all studies were approved by the Local Research Ethics Committee (Study 1 LREC2001/4/46; Study 2 LREC 2002/8/64; Study 3 LREC 09/S1101/54).

This study uses brain Fluid Attenuated Inversion Recovery (FLAIR), T1‐, T2‐, T2*‐, and DWI images with the sequence parameters summarized in Table S1 (supplementary material), acquired on a GE Signa Horizon HDx 1.5T clinical/research scanner (General Electric, Milwaukee, WI), equipped with a self‐shielding gradient set and manufacturer‐supplied eight‐channel phased‐array head coil. Calibration sequences, magnet shimming, and visual quality assurance were performed during each scanning session.

### 2.2 Assessment of the SVD imaging markers

**Figure 1:**
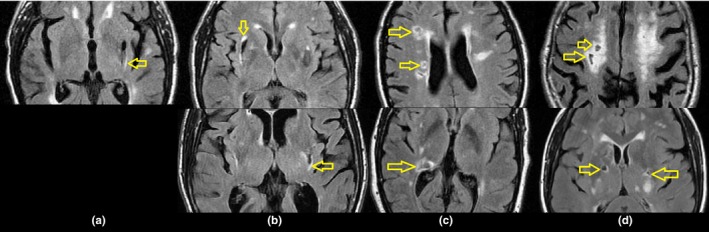
Cavitated white matter hyperintensities [cavitated white matter hyperintensities (WMH)]: in contact with lacunes but without overlap (a), partially overlapping with lacunes (b), surrounding (or completely overlapping with) cavities “in formation” (or possibly lacunes affected by partial volume effects due to Magnetic Resonance Images (MRI) slice thickness) (c), and totally overlapping with lacunes (d). The characteristic locations for each ‘class’ are shown

We followed published radiological and clinical definitions of RSSI, lacunes, WMH, old cortical infarcts, perivascular spaces, and brain microbleeds (Wardlaw, Smith, Biessels, et al., 2013). In addition, in the case of lacunes, when their intensity level on FLAIR was higher than that of CSF although still lower than the intensity level of the brain parenchyma, this was annotated for further consideration in the analyses under the assumption that these were lacunes “in formation” or “possibly lacunes” (probably confounded by MRI sequence parameters and partial volume effects due to the slice thickness). Also, those FLAIR/T2W hyperintense clusters that surrounded were partially bordering or contacted lacunes or “possibly lacunes”, separately delineated, and classed as “cavitated WMH”. This differentiation was done to allow analyzing the spatial distribution and extent of these WMH clusters seeking for a pattern in their location in the brain and with respect to the lacunes or “possibly lacunes”. These ‘cavitated WMH’ were visually classified following the visual rating scale proposed by Duering et al. (2013) as having contact only, partial overlap, or total overlap with the cavitated regions (Figure 1).

The RSSI, lacunes, WMH, and old cortical infarcts were all delineated using a multispectral thresholding‐based method (Hernandez, Ferguson, Chappell, & Wardlaw, 2010) implemented on a freely available tool (www.sourceforge.net/projects/bric1936) for their automatic identification, followed by manual editing using the region‐growing thresholding algorithm implemented in Analyze^™^10.0 (http://www.analyzedirect.com/Analyze/), as per Valdes Hernandez, Armitage, et al. (2015). To differentiate the lacunes and infarcts (new and old) from the surrounding brain on the FLAIR image, we considered a threshold equal to 3.5 SD in pixel intensity, respectively, below or above the mean of the fitted intensity distribution for the normal‐appearing white matter (Hernandez et al., 2010).

Perivascular spaces (Kwee & Kwee, 2007) were rated by an image analyst in basal ganglia and centrum semiovale following a visual rating scale validated in aging and stroke populations (Potter, Chappell, Morris, & Wardlaw, 2015). We computationally extracted all evidence of previous hemorrhagic episodes (Bradley, 1993), and using morphology analysis annotated the volume and count of the small and round T2*W hypointense clusters that adhered to the radiological definition of microbleeds (Cordonnier, Al‐Shahi Salman, & Wardlaw, 2007; Wardlaw, Smith, Biessels, et al., 2013). Visual scores for microbleeds and perivascular spaces done by an experienced (>20 years) neuroradiologist were available from 2/3 studies from which we extracted our sample. We evaluated the results of our computational assessment of microbleeds against the visual scores using robust (bootstrapped) Spearman correlation and the MATLAB Robust Correlation Toolbox (Pernet, Wilcox, & Rousselet, 2012). Using the same tool, and given the skewedness of the data, we recalculated the association after downweighting a percentage of marginal observations deviating from the median (i.e., bend correlation; Pernet et al., 2012). We evaluated the visual scores of perivascular spaces using the quadratic‐weighted kappa coefficient (http://vassarstats.net/kappa.html, © Richard Lowry 2001–2015, accessed on 02.03.2016) and marginal homogeneity analyses (Uebersax, 2006) as per Potter et al. (2015).

### 2.3 Lesion distribution quantification and analysis

We generated lesion probability distribution maps as described in Valdes Hernandez, Armitage, et al. (2015). To report and analyze the probability density distribution (PD) per location we used two complementary approaches: (1) we identified the regions of maximum PD and noted the location guided by a standard atlas (Duvernoy, 1999) and (2) we developed a computational ROI template by combining the subcortical structures extracted using FSL FIRST together with 2 × 2 × 2 mm region of interest (ROI) placed on five main white matter pathways selected from the Johns Hopkins University DTI white matter atlas (anterior horns of the lateral ventricles, optic radiations, centrum semiovale bordering the corona radiata, external capsules, and retrolentiform part of the internal capsules; Oishi et al., 2008), and then determined the PD at each computationally determined ROI. The PD in each voxel represents the proportion of the population with an acute small subcortical infarct, WMH, microbleed, old stroke lesion, cavitated WMH, or lacune involving that voxel. Thus, for example, if all patients had a WMH in the same voxel, the PD for WMH in that voxel would be 1; if half the population had a WMH in that voxel, the PD would be 0.5. These PD values were also adjusted by the number of individuals, within the population, that contributed data to the probability map. For example, whereas the whole stroke population had RSSI lesion(s), not all had old stroke lesions. Therefore, the PD of old stroke lesions was calculated considering only the number of patients who had them. The same happened for lacunes, cavitated WMH, and microbleeds, which were not present in all datasets.

### 2.4 Statistical Analysis

Imaging biomarker volumes were nonparametric, so descriptive data are reported as medians and interquartile range (IQR). We used the output of our automatic pipeline as input to IBM SPSS Statistics 21. We tested if imaging biomarker volumes grouped according to their location were significantly different from each other; the Wilcoxon matched‐pair rank/Mann–Whitney U tests were used to compare median differences, the Kendall's coefficient of concordance was used to quantify associations, and the Friedman's two‐way ANOVA by ranks/Kruskal–Wallis tests were used to compare distributions (i.e., frequency of occurrence). The chi‐squared goodness‐of‐fit test was used to compare the frequencies of lesions in each hemisphere. We considered *p* values <.05 as significant. All imaging biomarker volumes were standardized for head size by dividing the marker volume by the intracranial volume (ICV) for each patient. We were unable to exclude outliers due to the sensitivity of the functions to nonzero values. We were not always able to calculate significance due to small numbers.

## 3 Results

Image registration performed well for 192/194 stroke patients (99%); FLAIR data were incomplete/absent for 6 patients and corrupted with severe artifacts in 1, so the WMH volume and mapping of stroke patients is based on 188/194 patients and lacunes, old cortical strokes, and micro/macrohemorrhages on 187/194. Table 1 records descriptive statistics for all biomarkers.

**Table 1:** Frequency of occurrence (number and percent of patients in the sample) and median (and IQR) volumes of all the imaging biomarkers assessed

RSSI location	SVD imaging biomarker	Frequency [i.e., number of patients (%)]	Median volume or score (IQR)	% [median (IQR)] of total ICV
Total brain	RSSI	187/187 (100)	0.75 (0.84) ml	0.05 (0.06)
	WMH	187/187 (100)	16.82 (30) ml	1.18 (2.18)
	Cavitated WMH	64/187 (34)	1.15 (1.45) ml	8.02 (9.29) × 10^−4^
	Lacunes	87/187 (46)	0.17 (0.22) ml	1.15 (1.48) × 10^−4^
	Perivascular spaces	186/187 (99)	BG score: 2 (1)	Not applicable
			CS score: 3 (1)	
	Microbleeds	92/187 (49)	1 (3) (*n *= 187)	Not applicable
			3 (3) (*n *= 92)	
	Old cortical stroke lesions	41/187 (22)	0.54 (2.08) ml	3.84 (16.26) × 10^−4^
Left hemisphere	RSSI	97/187 (52)	0.71 (1.11) ml	0.05 (0.08)
	WMH	97/97 (100)	12.61 (25.30) ml	0.94 (1.95)
	Cavitated WMH	32/97 (33)	1.19 (1.57) ml	8.65 (11.92) × 10^−4^
	Lacunes	44/97 (45)	0.17 (0.20) ml	1.30 (1.40) × 10^−4^
	Perivascular spaces	97/97 (100)	BG score: 2 (2)	Not applicable
			CS score: 3 (1)	
	Microbleeds	50/97 (52)	1 (3) (*n *= 97)	Not applicable
			3 (3) (*n *= 50)	
	Old cortical stroke lesions	21/97 (22)	0.70 (3.52) ml	4.69 (27.00) × 10^−4^
Right hemisphere	RSSI	78/187 (42)	0.79 (0.82) ml	0.5 (0.06)
	WMH	78/78 (100)	23.09 (28.24) ml	1.64 (2.09)
	Cavitated WMH	29/78 (37)	1.06 (1.42) ml	7.04 (8.19) × 10^−4^
	Lacunes	37/78 (47)	0.17 (0.35) ml	1.15 (2.11) × 10^−4^
	Perivascular spaces	77/78 (99)	BG score: 2 (1)	Not applicable
			CS score: 3 (1)	
	Microbleeds	38/78 (49)	1 (2) (*n *= 78)	Not applicable
			2 (4) (*n *= 38)	
	Old cortical stroke lesions	17/78 (22)	0.67 (1.81) ml	4.61 (11.80) × 10^−4^
Both hemispheres	RSSI	5/187 (3)	1.77 (2.81) ml	0.14 (0.02)
	WMH	5/5 (100)	60.47 (73.52) ml	4.98 (4.28)
	Cavitated WMH	2/5 (40)	1.25 ml	7.78 × 10^−4^
	Lacunes	4/5 (80)	0.17 (0.35) ml	1.18 (2.08) × 10^−4^
	Perivascular spaces	5/5 (100)	BG score: 2 (2)	Not applicable
			CS score: 2 (2)	
	Microbleeds	3/5 (60)	2	Not applicable
	Old cortical stroke lesions	1/5 (20)	0.32 ml	1.93 × 10^−4^
Cerebellum/ brainstem	RSSI	7/187 (4)	0.33 (0.20) ml	0.02 (0.02)
	WMH	7/7 (100)	19.69 (16.66) ml	1.27 (1.19)
	Cavitated WMH	1/7 (14)	0.49 ml	3.14 × 10^−4^
	Lacunes	2/7 (28)	0.09 ml	0.61 × 10^−4^
	Perivascular spaces	7/7 (100)	BG score: 2 (1)	Not applicable
			CS score: 2 (2)	
	Microbleeds	1/7 (29)	1	Not applicable
	Old cortical stroke lesions	2/7 (28)	0.61 ml	4.92 × 10^−4^

RSSI, recent small subcortical infarct; SVD, small vessel disease; IQR, interquartile range; ICV, intracranial volume; WMH, white matter hyperintensities.

The median volumes refer only to those patients who had the biomarker, not to the total sample. The frequency of occurrence reflects the incidence of the specific biomarker with respect to the location of the RSSI. Hence, the numerator shows the number of patients who had the biomarker in the location/hemisphere specified and the denominator the number of patients who had the RSSI on that same location/hemisphere. For the perivascular spaces, median (IQR) scores in the basal ganglia (BG score) and centrum semiovale (CS score) are given.

As detailed in the sections below, there was no association between the volume of the RSSI and the volume of any imaging biomarker, between the location of the RSSI and the location of any of the imaging biomarkers, or between the prevalence of any imaging biomarker (i.e., count) and the volume of the RSSI.

### 3.1 RSSI volume and distribution

**Figure 2:**
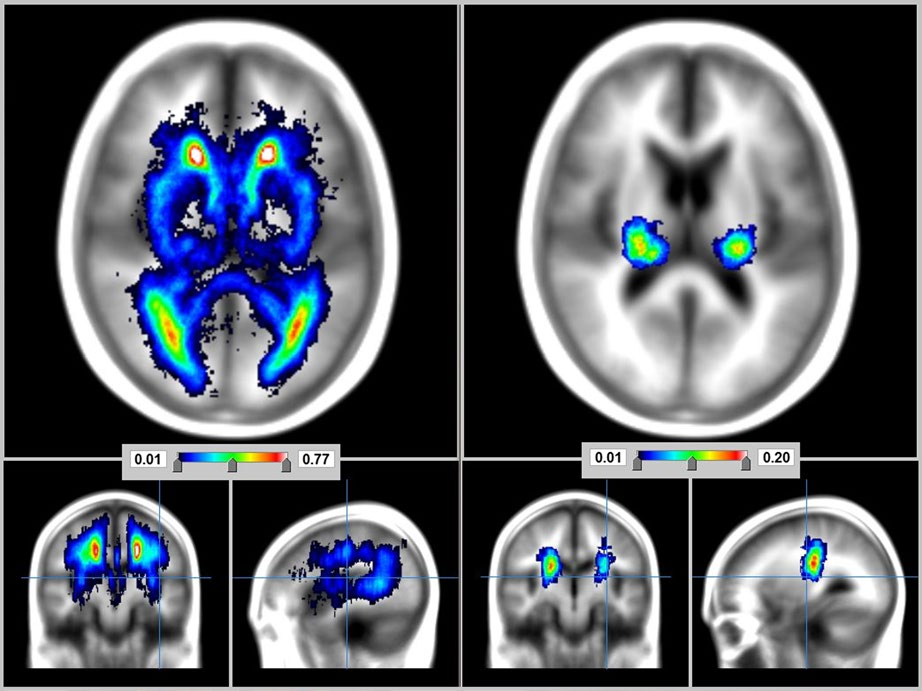
Probability distribution maps of white matter hyperintensities (WMH) (left) and recent small subcortical infarcts (RSSI) (right) on the sample

51.9% (*n *= 97) of the population had the RSSI in the left hemisphere, 41.7% (*n *= 78) on the right, 2.7% (*n *= 5) in both hemispheres, and 3.7% (*n *= 7) in the cerebellum, midbrain, or brainstem. There was no significant left‐right difference in RSSI frequency (Friedman's two‐way ANOVA and Kendall's coefficient of concordance *p *= .10). The median percentage volume of the RSSI in ICV was 5.33 × 10^−4^ (IQR = 2.7 × 10^−4^–8.9 × 10^−4^). There was no significant right‐left difference in RSSI volumes (Wilcoxon signed rank *p *= .16), with spatial prevalence already reported in Valdes Hernandez, Maconick, et al. (2015). RSSI was distributed slightly more widely on the left (Figure 2). The median PD of RSSI did not differ significantly between left and right hemispheres (related samples Wilcoxon signed rank *p *= .058) (Figure 2).

### 3.2 Imaging biomarkers prevalence by RSSI hemisphere

**Figure 3:**
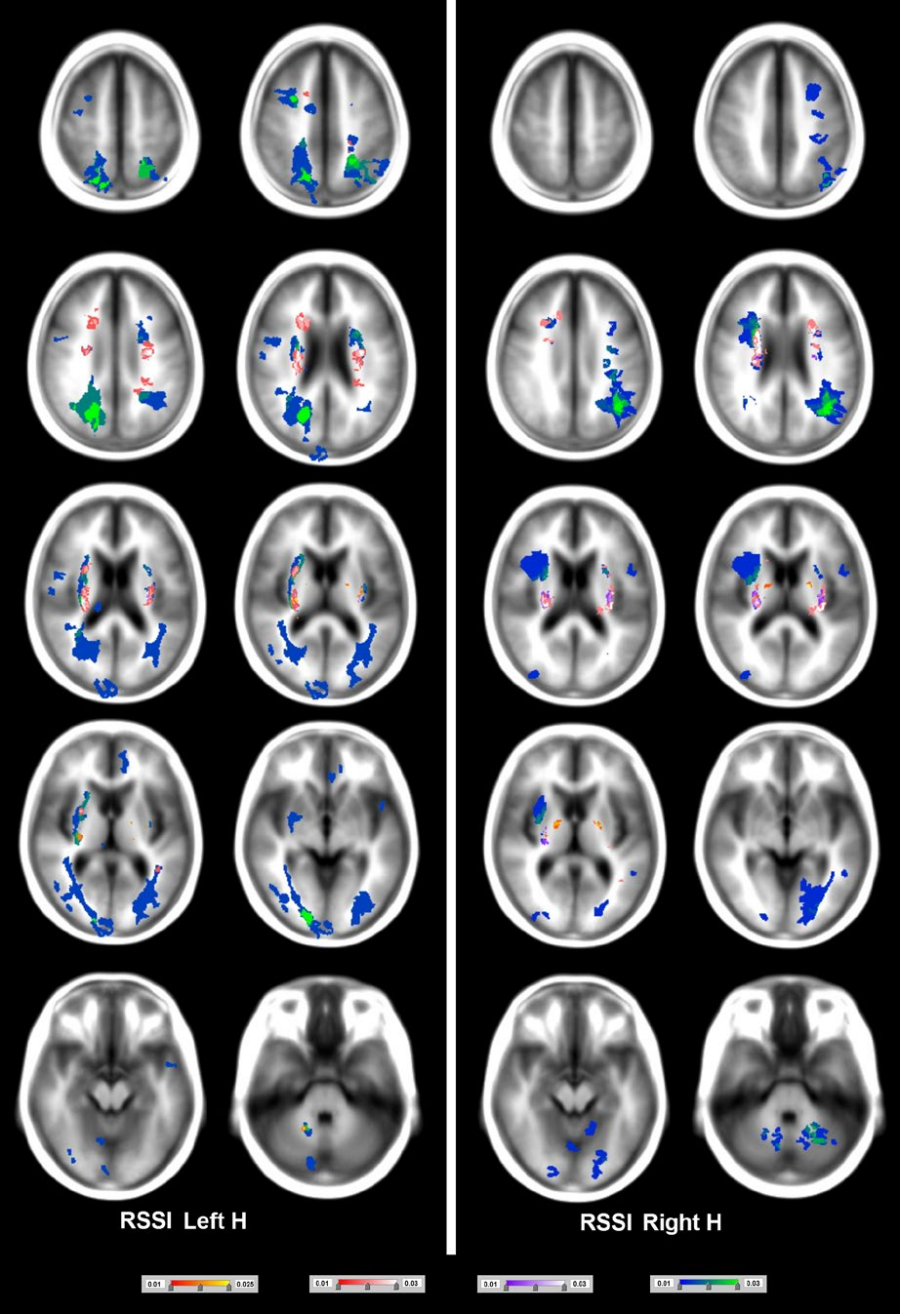
Probability distribution of micro/macrohemorrhages (scale from red to yellow), cavitated white matter hyperintensities (scale from magenta to white), lacunes (scale from purple to white), and old cortical infarcts (scale from blue to green). The minimum probability density was in all cases 0.01/mm^3^, and the maximum was 0.025/mm^3^ (for micro/macrohemorrhages) or 0.03/mm^3^ (for the rest). Axial slices at the left‐hand side correspond to maps of biomarkers that occurred when the recent small subcortical infarct (RSSI) was at the left hemisphere, and those at the right‐hand side correspond to maps of biomarkers that appeared when the RSSI was at the right hemisphere

The most common biomarker was WMH, found in 100% of the patients, followed by perivascular spaces (present in all datasets except one). WMH and RSSI were predominant in the cerebral hemispheres (RSSI on 96% of the sample and WMH on 100% of the sample) as opposed to the midbrain/brainstem (both on only 4% of the total sample) (Kruskal–Wallis *p *= .03 for WMH and *p *= .005 for RSSI) (Table 1, Figure 2). The volumes of the other biomarkers did not significantly differ when analyzed by RSSI location (i.e., left hemisphere, right hemisphere, brainstem/midbrain, or bilateral) (Kruskal–Wallis .33 < *p *< .99) (Figure 3).

### 3.3 WMH incidence, volume, and distribution

WMH were distributed symmetrically between right and left sides of the brain. The median and spatial PD of WMH occurrence for the left and right hemispheres did not differ significantly (Wilcoxon *p *= .47, Friedman's two‐way ANOVA, and Kendall's coefficient of concordance *p *= .74). Of note, the WMH PDs were relatively lower in the structures where the RSSI had the highest PDs (e.g., 0.1–0.15 at the posterior limb of the internal capsule adjacent to the lateral ventricles) than in other cerebral white matter regions (e.g., 0.77/mm^3^ in anterior hemispheric white matter where WMH were most frequent, 0.53–0.57/mm^3^ elsewhere in centrum semiovale, and 0.5/mm^3^ in optic radiation) (Figure 2).

**Figure 4:**
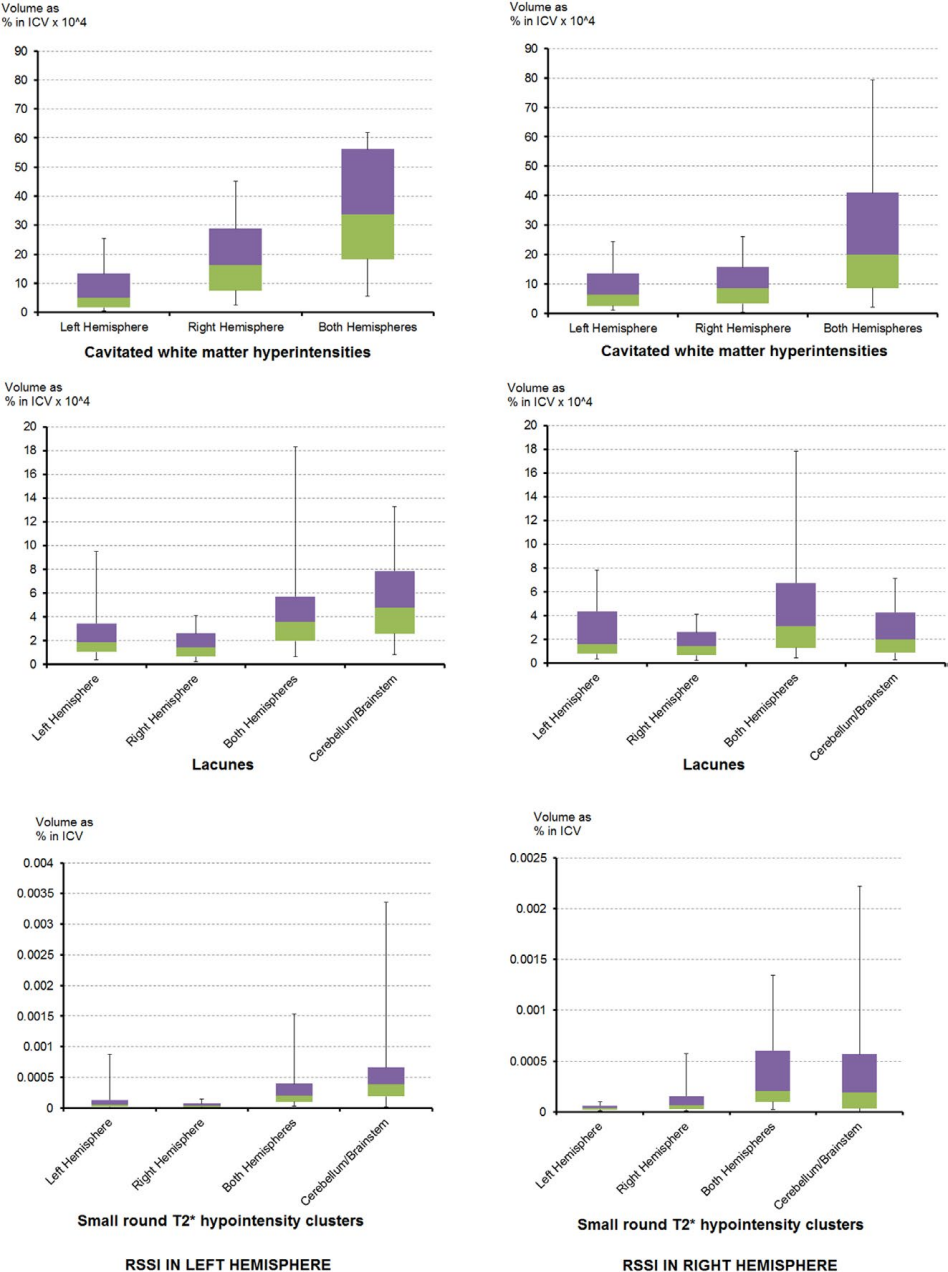
Box plots of the volumetric distribution per hemisphere of lacunes, cavitated white matter hyperintensities, and microbleeds in the sample (the latter calculated as volume of the small round T2* hypointensity clusters computationally identified). ICV, intracranial volume

Thirty‐two of ninety‐seven (33%) patients with the RSSI on the left and 29/78 (37%) of those with the RSSI on the right had WMH partially or completely surrounding the lacunes (cavitated WMH). The percent of patients who had cavitated WMH per location (i.e., left/right hemispheres, bilateral and cerebellum, and brainstem) was similar regardless of the hemisphere where the RSSI was (Table 2). There was no significant difference between the volumes of cavitated WMH in subjects who had them only in the left and those who had them only in the right hemisphere (Wilcoxon's *p *= .53) regardless of the RSSI location (Figures 3 and 4). Cavitated WMH were mainly found bilaterally (median percentage of ICV: 8.02 × 10^−4^ in total and 7.78 × 10^−4^ when found bilaterally) (Table 1).

**Table 2:** Location of each biomarker in relation to the hemisphere of the supratentorial RSSI

Biomarker and RSSI hemisphere	Location of biomarker	Number of patients (%)	Median number of occurrences per patient
Lacunes, RSSI in left hemisphere	Left hemisphere	14/97 (14.4)	1
	Right hemisphere	9/97 (9.3)	1
	Cerebellum/brainstem	4/97 (4.1)	4
	Bilateral	17/97 (17.5)	3
Lacunes, RSSI in right hemisphere	Left hemisphere	9/78 (11.5)	2
	Right hemisphere	7/78 (9.0)	1
	Cerebellum/brainstem	6/78 (7.7)	2
	Bilateral	15/78 (19.2)	3
Cavitated WMH, RSSI in left hemisphere	Left hemisphere	11/97 (11.3)	Not counted, volume of the hyperintense cluster noted
	Right hemisphere	9/97 (9.3)	
	Cerebellum/brainstem	2/97 (2.1)	
	Bilateral	10/97 (10.3)	
Cavitated WMH, RSSI in right hemisphere	Left hemisphere	9/78 (11.5)	Not counted, volume of the hyperintense cluster noted
	Right hemisphere	5/78 (6.4)	
	Cerebellum/brainstem	1/78 (1.3)	
	Bilateral	14/78 (18.0)	
Microbleeds, RSSI on left hemisphere	Left hemisphere	13/97 (13.4)	1
	Right hemisphere	8/97 (8.2)	1
	Cerebellum/brainstem	13/97 (13.4)	6
	Bilateral	24/97 (24.7)	3
Microbleeds, RSSI on right hemisphere	Left hemisphere	5/78 (6.4)	1
	Right hemisphere	10/78 (12.8)	1
	Cerebellum/brainstem	15/78 (19.2)	5
	Bilateral	15/78 (19.2)	2
Old cortical stroke lesion, RSSI on left hemisphere	Left hemisphere	8/97 (8.2)	1
	Right hemisphere	5/97 (5.2)	
	Cerebellum/brainstem	6/97 (6.2)	
	Bilateral	2/97 (2.1)	
Old cortical stroke lesion, RSSI on right hemisphere	Left hemisphere	2/78 (2.6)	1
	Right hemisphere	2/78 (2.6)	
	Cerebellum/brainstem	12/78 (15.4)	
	Bilateral	1/78 (1.3)	

RSSI, recent small subcortical infarct; WMH, white matter hyperintensities.

Cavitated WMH clusters were present on 32 patients with the RSSI in the left and 29 patients with the RSSI in the right. The proportion of cavities completely surrounded by these clusters as opposed to those “at the edge” was not uniform among these two groups of patients: 43% of the left‐sided RSSI versus 37% of the right‐sided RSSI had cavitated WMH clusters completely surrounding the cavities (complete overlap); and 53% of the left‐sided RSSI versus 30% of the right‐sided RSSI had a partial overlap. In only one patient a WMH cluster was in contact with the lacune without having any overlap (Figure 1a). However, a distributional pattern of the appearance of these clusters, in relation to the lacunes, emerged from the analysis. In general, the WMH clusters that partially overlapped with lacunes were found almost exclusively in the external capsule, on the inferior junction between the internal and external capsules (Figure 1b) or in the thalamus. WMH clusters that totally overlapped with “lacunes in formation” were always in the periventricular region (Figure 1c), whereas WMH clusters that totally overlapped with well‐defined lacunes were in the deep white matter or junctions between deep white and grey matter (either cortical or deep) (Figure 1d).

### 3.4 Lacune count, volume, and distribution

Lacunes were identified in 87/187 patients, from which 54 had two or more lacunes (45/54 had lacunes in both hemispheres). Lacunes were in the left hemisphere in 23/187 patients, right hemisphere in 16/187 patients, and in the brainstem in 10/187 patients (Tables 1 and 2). Of those with the RSSI in the left hemisphere, 45.4% had lacunes versus 47.4% when the RSSI was on the right (Table 1). There was no significant difference between the number of patients who had lacunes on the left and those who had them on the right regardless of the RSSI location (Wilcoxon's *p *= .4 if RSSI was on the left and *p *= .9 if RSSI was on the right). The volumes of all biomarkers (RSSI, WMH, old cortical stroke lesions, and micro/macrohemorrhages) in those with lacunes were significantly larger than the volumes in those without lacunes (Mann–Whitney U from *p *< .001 to *p *= .04).

The median volume of lacunes was 0.17 ml (IQR 0.22 ml, Table 1). There was no difference in lacunar volume between left and right hemispheres (Wilcoxon's *p *= .47) (Figure 4). Lacunes prevailed in the anatomical regions adjacent to those where RSSI were most frequent, with PD approximately 0.05/mm^3^. However, lacunes were also present in deep grey matter regions distant from the RSSI (e.g., caudate (PD 0.01/mm^3^), hippocampus (PD 0.01/mm^3^), and thalamus (PD 0.005/mm^3^)). Lacunes “in formation” were almost exclusively found adjacent to the anterior and posterior horns of the lateral ventricles, surrounded by WMH.

### 3.5 Perivascular spaces visual scores

All patients except one had perivascular spaces, with load balanced in both hemispheres. The scores in the left basal ganglia for those with the RSSI in the left were as follows: 28/97 (29%) patients with 1, 39/97 (40%) with 2, 19/97 (20%) with 3, and 11/97 (11%) with 4. Two of the patients with score 1 in the left basal ganglia had score 2 in the right. In the left centrum semiovale, the scores for left‐sided RSSI patients were as follows: 11/97 (11%) patients with 1, 34/97 (35%) patients with 2, 38/97 (39%) patients with 3, and 12/97 (12%) patients with 4. Only one patient had unbalanced scores in the centrum semiovale (1 in the left and 2 in the right). Patients with the RSSI in the right had the same scores in the left and right centrum semiovale: 6/78 (8%) patients with 1, 25/78 (32%) patients with 2, 31/78 (40%) patients with 3, and 10/78 (13%) patients with 4. Although five right‐sided RSSI patients had slightly unbalanced PVS load in the basal ganglia (i.e., differing by 1 score), the proportion of patients in each score was very similar to that of the left‐sided RSSI group. Similar scores were given by an experienced neuroradiologist in the subsample for which these were available (*n *= 105). For the basal ganglia, the interrater kappa was 0.8 (95% confidence interval (CI) [0.68 0.92]), and for the centrum semiovale 0.64 (95%CI [0.43 0.84]). Marginal homogeneity analyses showed a difference of 10% between raters in thresholding categories 1 and 2, but this tendency was consistent (i.e., in the same direction).

### 3.6 Microbleeds count, volume, and distribution

The frequency of occurrence of small spherical clusters of T2*W hypointensities classed as microbleeds is summarized in Table 3. Visual assessment was associated with the computational assessment: Spearman ρ = 0.56, *p *< .0001, 95%CI [0.33 0.75] for the left hemisphere, and Spearman ρ = 0.60, *p *< .0001, 95%CI [0.32 0.79] for the right hemisphere. Bootstrapped correlations yielded similar results. The computational method only recognized hypointensities below half of the median intensity level of the normal‐appearing white matter, missing microbleeds affected by partial volume effects. The number of microbleeds visually counted was always higher than the number computationally determined on patients with >5 of them.

**Table 3:** Frequency of occurrence (i.e., number of patients) of small round T2*W hypointense clusters (i.e., computationally determined as microbleeds) in the sample

Hemisphere of supratentorial RSSI	Number of Microbleeds	Microbleeds in the left hemisphere		Microbleeds in the right hemisphere	
		Frequency	%	Frequency	%
Left hemisphere	0	49	51.6	55	57.9
	1	22	23.2	15	15.8
	2	16	16.8	11	11.6
	3	2	2.1	4	4.2
	4	3	3.2	3	3.2
	≥5	3	3.3	7	7.5
Right hemisphere	0	52	67.5	49	63.6
	1	12	15.6	10	13.0
	2	5	6.5	10	13.0
	3	4	5.2	2	2.6
	4	1	1.3	2	2.6
	≥5	3	3.9	4	5.2

RSSI, recent small subcortical infarct.

Patients with microbleeds in the cerebellum or midbrain showed the greatest median frequency of microbleeds (approximately 6) (Table 2). Microbleeds were most likely to be found bilaterally per patient (Table 2) and in the sample (Table 3 and Figures 3 and 4), most commonly in the basal ganglia and in the corticomedullary junctions of white matter. Patients with microbleeds, in general, had significantly larger volume of lacunes (*p *< .001) and WMH (*p *= .001) than patients without, before, and after correcting for head size.

### 3.7 Old cortical stroke lesions count, volume, and distribution

Patients with a left‐sided RSSI (21/97, 21.7%) and right (17/78, 22%) had old cortical stroke lesions (Table 1). There was no difference in old stroke lesion volume between left and right hemispheres in those with a left‐sided RSSI (Wilcoxon's *p *= .72). In those who had the RSSI on the right (*n *= 17), two patients had an old cortical stroke lesion in the right hemisphere, two in the left, with the majority in the cerebellum/brainstem (*n *= 12) (Table 2). When the RSSI was in the left hemisphere, old stroke lesions affected mainly the optic radiations on the posterior cerebral artery territory, although small cortical lesions occurred on the left middle cerebral artery and right anterior cerebral artery territories (Figure 3). The maximum PD in regions affected by old cortical strokes was 0.03/mm^3^ (Figure 3). Total volumes of lacunes and WMH were significantly larger in patients with versus patients without old cortical stroke lesions (Mann–Whitney U *p *= .004 and *p *= .04, respectively), before and after correcting for head size.

## 4 Discussion

### 4.1 Main findings

The lack of association between the volume and location of the RSSI and the burden and location of any of the SVD markers assessed suggests that the latter appear similarly in ipsi‐ and contralateral brain hemispheres and that any problems with the vasculature are not specific to a single hemisphere in patients with a RSSI and without evidence of a previous brain trauma, in agreement with other studies (Wardlaw, Smith, Biessels, et al., 2013). The most common regions for RSSI, lacunes, and small T2*W hypointense clusters were the deep grey matter structures and their vicinities. A study examining the distribution of brain microbleeds and lacunes also found that lacunes were mainly located in and around deep grey matter structures (Lee et al., 2004). But different from the present analysis reported that the most common region for microbleeds was at the cortico–subcortical junction, attributing it to an increased susceptibility for microbleeds in this region (Lee et al., 2004).

### 4.2 RSSI hemisphere and location

RSSI was found almost exclusively in the posterior limb of the internal capsule and neighboring white and deep grey matter areas: posterior putamen, globus pallidus, and anterior thalamus; coincident with the region assumed to be crossed by the sensory and motor pathways as published previously (Valdes Hernandez, Maconick, et al., 2015). We also found a balance between the number of patients who had the RSSI in the left and right hemisphere, in agreement with another study (Mead, Lewis, Wardlaw, Dennis, & Warlow, 2000). However, these findings may not be conclusive as, although we were unable to show greater incidence of RSSI on the left, a higher‐powered study with the same variance might result in significance. In fact, few studies have reported left hemisphere strokes being more common (Foerch, Misselwitz, Sitzer, Berger, & Steinmetz, 2005; Hedna et al., 2013). A study found that the left cerebral vessels of patients with hypertension tended to be weaker and that there were differences in the intima–media thickness of the common carotid arteries, but was unable to prove if this was causally associated with stroke (Rodriguez Hernandez et al., 2003). Another study (Hedna et al., 2013) found large‐vessel events were more prevalent on the left as well as a predisposition for the RSSI to occur on the left. Although this indicates a potential weakness in the left hemisphere it has been noted that this may not reflect small vessels. Our sample only comprised individuals presenting with symptoms. Broca's and Wernicke's speech areas are located in the left hemisphere for about 95% of right handers, but about 70% of left handers (Knecht et al., 2000). It is possible that the stroke could be less noticeable in the right hemisphere perhaps as the speech is less likely to be affected, thus indicating that maybe a percentage of RSSI that occur in the right hemisphere is disregarded and, therefore, unreported.

Only few patients had infratentorial RSSI. Diffusion weighted imaging slice thickness (5 mm) and time elapsed between the stroke onset and the MRI scan (from 1 to 4 weeks) could be causes of the low proportion of patients with positive DWI infarct in this region compared to those with the RSSI in the supratentorium. A study comparing 5‐mm spatial resolution DWI with 3‐mm DWI in the detection of acute infratentorial infarction concluded that the 3‐mm DWI adds sensitivity compared to the conventional 50mm DWI (reportedly 81.1% vs. 94.6%) with only a small reduction (2.3%) in specificity (Entwisle, Perchyonok, & Fitt, 2016). Another study that examined the size and evolution of acute brainstem ischemic lesions reports a decrease over time in the volume of the infratentorial stroke lesion detected in DWI with a mean shrinking factor of 3.3 between the scan acquired at 24 hr from the stroke onset and the follow‐up DWI acquired at a median of 4.8 months later (Fitzek et al., 2001).

### 4.3 The imaging biomarkers

The cerebellum/midbrain/brainstem was the least common area for lacunes and WMH. A longitudinal study on the progression of lacunes in patients with cerebral autosomal‐dominant arteriopathy with subcortical infarcts and leukoencephalopathy (Duering et al., 2013) found a prevalence of lacunes at the edge of WMH along the anatomical course of the perforating vessels. We found lacunes to be with similar frequency both at the edge of WMH and/or totally surrounded by WMH (irrespective of the RSSI site). Hypointense regions on FLAIR with morphological characteristics identical to those from lacunes, but with intensity levels higher than that of CSF, which we called cavities “in formation”, appeared always surrounding the ventricles, although not necessarily on the same side as the index RSSI.

The prevalence of microbleeds was greater in our sample (computationally found in 49% of the sample) than in a nonstroke population of similar mean age (12%; Sveinbjornsdottir et al., 2008). This is to be expected as it has been reported that the incidence of brain microbleeds increases in the presence of lacunar infarction and cardiovascular risk factors (Gao, Wang, & Zhang, 2008; Kato, Izumiyama, Izumiyama, Takahashi, & Itoyama, 2002). Our reported prevalence was lesser to that of a study comparing the distribution of lacunes and microbleeds which found a microbleed prevalence of 55.8% in hypertensive patients (Sun et al., 2009), and to other studies on populations with intracerebral hemorrhages: 66% (Lee, Kim, & Roh, 2006) and 68% (Jeon et al., 2007). Multiple microbleeds increase the risk of future ischemic events (Cordonnier et al., 2007). However, we did not find an association between number of microbleeds and indicators of possible previous ischemic episodes (lacunes and old stroke lesions).

### 4.4 Strengths and limitations

We used imaging data from a large population of stroke patients enrolled in three stroke studies covering an 11‐year period, and including as many patients as possible with acute lacunar stroke symptoms and a DWI‐visible lesion to help ensure correct identification of the index stroke lesion. We also assessed in detail most of the main SVD imaging biomarkers using validated semiautomatic methods. We used a study‐specific age‐ and population‐relevant brain template to optimize the image registration, reduce spatial distortion, minimize bias of lesion size or location, and used volumetric measurements done in native space and corrected by ICV to avoid lesion distortion in standard space and account for differences in overall head size across patients.

Despite being one of the strengths of our study to collectively look at all imaging biomarkers of SVD to gain wider understanding of the distributional association of its manifestations (Wardlaw, Smith, Biessels, et al., 2013), our results do not reflect the true burden of SVD markers. The long‐term appearance of the symptomatic small subcortical infarcts varies: up to 10% disappear completely, between 30 and 95% cavitate to form a lacune, and the rest retain an appearance identical to a WMH (Loos, Staals, Wardlaw, & van Oostenbrugge, 2012; Moreau et al., 2012; Potter et al., 2010). Some other features like cortical microinfarcts and tissue changes in the normal‐appearing white matter cannot be identified on standard MRI (Gouw et al., 2011) at 1.5T. Also, the size of lacunes change with time with newer lacunes tending to be larger due to an acute swelling.

Despite the advances in technology, it is still difficult to differentiate between large perivascular spaces and lacunes and there is a small degree of chance that some of the segmented lacunes are, in fact, perivascular spaces. This could potentially confound part of our results. Although an experienced neuroradiologist advised on the more difficult cases, an element of uncertainty exists. Issues exist with microbleeds too. Accurate assessment of microbleeds is known to be hampered by several caveats (Cordonnier et al., 2007). Visual interobserver agreements report median kappa in the range between 0.44 and 0.78 (Cordonnier et al., 2007), and the computational methods existent up‐to‐date have reported degrees of error ranging from 20% to 30% (Barnes et al., 2011; Seghier et al., 2011). Our computational assessment is not exempt of errors. What computationally is identified as small, circular, T2*W hypointense foci does not always represent a microbleed and could alternatively be a vessel calcification, which can strongly mimic the microbleeds’ appearance (Charidimou, Krishnan, Werring, & Rolf Jager, 2013). On the other hand, microbleeds assessment could be inaccurate when these appear pale or in a position susceptible to partial volume effects (Cordonnier et al., 2007). In addition, computed tomography scans were not available to exclude calcifications and neither were follow‐up scans to evaluate if the “uncertain microbleeds” mature into “certain microbleeds”.

Our analyses were not adjusted for risk factors and some patients did have hypertension and previous history of cardiovascular disease. Cardiovascular risk factors such as hypertension and previous cardiovascular events can hasten the spread of SVD. Another strong risk factor is age. Our patients had a wide range in terms of age. It has been reported that the prevalence of silent infarcts, a defining SVD feature, in 60 year olds was around 6%, rising to 28% in those at age 80 (Thompson & Hakim, 2009).

It is difficult to ascertain the extent to which SVD contributes to the location of the biomarkers seen in our patients as other neurological diseases share some of the same biomarkers as SVD. For example, both Alzheimer's disease and intracerebral hemorrhage are known to be associated with the presence and prevalence of brain microbleeds (Charidimou et al., 2013), as well as aging and hypertension. Whereas cerebral amyloid angiopathy pathology is said to be more likely to be associated with a lobar distribution of microbleeds; hypertension and lipohyalinosis, both related to SVD, are said to be more likely to be associated with brain microbleeds in the deep structures (e.g., basal ganglia).

### 4.5 Future directions

The full link between lesion location and clinical correlates has not yet been elucidated; brain networks are widespread and still not fully understood. Although there is a large literature base, most studies do not recognize the interplay of the different biomarker pathologies and focus on specific aspects of SVD. A potential direction that could be taken is to investigate the association between the severity of clinical symptoms and the location of SVD biomarkers. Some studies have taken this direction but most tend to investigate the effect of the symptomatic stroke (O'Sullivan, 2010).

Lacunes or cavities “in formation” fully overlapping with WMH clusters also appear on other pathologies, for example, multiple sclerosis. Coincidently, they are located almost exclusively adjacent to the horns of the lateral ventricles. This could suggest that these formations may have a distinctive etiology. However, their frequency of occurrence is low. Large longitudinal brain databases are necessary to study their progression and discern whether or not they are really lacunes in formation or other manifestation of white matter disease.

We found that a small percentage of patients had lacunes coexistent with old cortical stroke lesions. It would be interesting to examine if the aggregate of different biomarkers had an effect on the severity of the lacunar stroke measured by clinical symptoms and functional outcome severity in a higher‐powered age‐controlled study. Future studies should also look to examine the association between different vascular territories and the distribution of imaging biomarkers. These would help provide clues to developing a comprehensive understanding of the pathophysiology of SVD.

## Conflicts of interest

None declared.
