# Supplementary material for: Interhemispheric characterization of small vessel disease imaging markers after subcortical infarct
Source: Brain Behav. 2016 Nov 3;7(1):e00595. doi: 10.1002/brb3.595 (PMC5256179; doi:10.1002/brb3.595)
Supplement: Supplementary file 1 [file BRB3-7-e00595-s001.docx]

**Inter-hemispheric characterisation of small vessel disease imaging markers after subcortical infarct**

**Supplementary material**

Table S1. MRI sequence details of the primary studies that contributed data to this work.

| Study | Stroke Study 1 | Stroke Study 2 | Stroke Study 3 |
| --- | --- | --- | --- |
| TR/TE/TI (ms) T1W | 440/9 | | 9.7/3.984/500 |
| TR/TE (ms) DT-MRI | 6300/106.13 | 6300/107.64 | 10000/95.5 |
| TR/TE (ms) T2*W | 2000/9.912 | 620/15 | 625/15 |
| TR/TE/ TI (ms) FLAIR | 10002/147/ 2500 | 9002/147/2200 | 9000/140/2200 |
| Pixel bandwidth (KHz) | 125 (T1W)  81.38 (T2*W)  122.07(FLAIR) | 125 (T1W)  97.656(T2*W)  122.07(FLAIR) | 15.63 (T1-W,FLAIR)  12.5 (GRE) |
| Matrix | 256x256 | 256x192 | 256x216 (T1W)  128x128 (DTI)  384x224 (T2*W, FLAIR) |
| No. slices | 19 | 20 | 256 (T1W)  28 (FLAIR,T2*W, T2W) |
| Slice thickness (mm) | 5 | 5 | 1.02 (T1W)  5 (DTI,T2*W, FLAIR) |
| Inter-slice gap (mm) | 1.5 | 1.5 | 1 |
| Voxel size (mm^3^) | 0.94x0.94x6.5 | 0.94x0.94x6.5 | 1.02x0.9x1.02 (T1W)  0.47x0.47x6 (T2-W,FLAIR, GRE) |
